# Deep learning assisted quantitative assessment of histopathological markers of Alzheimer’s disease and cerebral amyloid angiopathy

**DOI:** 10.1186/s40478-021-01235-1

**Published:** 2021-08-21

**Authors:** Valentina Perosa, Ashley A. Scherlek, Mariel G. Kozberg, Lindsey Smith, Thomas Westerling-Bui, Corinne A. Auger, Serge Vasylechko, Steven M. Greenberg, Susanne J. van Veluw

**Affiliations:** 1 J. Philip Kistler Stroke Research Center, Department of Neurology, Massachusetts General Hospital, Harvard Medical School, Cambridge Str. 175, Suite 300, Boston, MA 02114 USA; 2 Department of Neurology, Otto-Von-Guericke University, Magdeburg, Germany; 3 Rush Alzheimer Disease Center, Rush University Medical Center, Chicago, IL USA; 4 MassGeneral Institute for Neurodegenerative Disease, Massachusetts General Hospital, Charlestown, MA USA; 5 Aiforia Inc, Cambridge, MA USA; 6 Computational Radiology Laboratory, Boston Children’s Hospital, Harvard Medical School, Boston, MA USA

**Keywords:** Alzheimer’s disease, Artificial intelligence, Cerebral amyloid angiopathy, Deep learning, Histopathology

## Abstract

Traditionally, analysis of neuropathological markers in neurodegenerative diseases has relied on visual assessments of stained sections. Resulting semiquantitative scores often vary between individual raters and research centers, limiting statistical approaches. To overcome these issues, we have developed six deep learning-based models, that identify some of the most characteristic markers of Alzheimer’s disease (AD) and cerebral amyloid angiopathy (CAA). The deep learning-based models are trained to differentially detect parenchymal amyloid β (Aβ)-plaques, vascular Aβ-deposition, iron and calcium deposition, reactive astrocytes, microglia, as well as fibrin extravasation. The models were trained on digitized histopathological slides from brains of patients with AD and CAA, using a workflow that allows neuropathology experts to train convolutional neural networks (CNNs) on a cloud-based graphical interface. Validation of all models indicated a very good to excellent performance compared to three independent expert human raters. Furthermore, the Aβ and iron models were consistent with previously acquired semiquantitative scores in the same dataset and allowed the use of more complex statistical approaches. For example, linear mixed effects models could be used to confirm the previously described relationship between leptomeningeal CAA severity and cortical iron accumulation. A similar approach enabled us to explore the association between neuroinflammation and disparate Aβ pathologies. The presented workflow is easy for researchers with pathological expertise to implement and is customizable for additional histopathological markers. The implementation of deep learning-assisted analyses of histopathological slides is likely to promote standardization of the assessment of neuropathological markers across research centers, which will allow specific pathophysiological questions in neurodegenerative disease to be addressed in a harmonized way and on a larger scale.

**Supplementary Information:**

The online version contains supplementary material available at 10.1186/s40478-021-01235-1.

## Introduction

The evaluation of histopathological brain sections is of great importance in neurodegenerative disease, not only to obtain a definite diagnosis but also to study disease-specific pathophysiological mechanisms. Alzheimer’s disease (AD) and cerebral amyloid angiopathy (CAA) are common neurodegenerative diseases in the elderly which are both characterized by accumulation of amyloid-β (Aβ) in the form of parenchymal plaques [2] and vascular Aβ in the walls of the small cortical and leptomeningeal blood vessels [10]. Other histopathological observations, such as iron deposition (indicative of hemorrhage and siderosis) [7, 9], reactive astrocytes and microglia (indicative of neuroinflammation) [19], and fibrin accumulation in the walls of small vessels and surrounding cells (indicative of blood–brain-barrier [BBB] leakage) [12, 16] are also crucial to understand complex disease mechanisms. These markers have so far mainly been quantified using visual semiquantitative scores [11] or manual count [15, 32]. Even when the scores are well-defined and their reliability assessed by inter-rating [32], they remain subjective and time-consuming to standardize across centers [24]. Moreover, the lack of continuous measures can prevent investigators from using more complex statistical models to better test hypotheses regarding disease pathophysiology. Several (semi)automated assessments have been developed to overcome these issues, but they mainly rely on pre-defined ranges of pixel color and intensity, such as red–green–blue (RGB) or hue-saturation-value (HSV) ranges [25, 30]. This makes them subject to the variability of fixation, slice thickness, stain intensity, and laboratory of origin. Furthermore, they do not allow the recognition and count of specific objects (e.g. detection of specific cells, such as individual astrocytes). Software that allow the characterization of specific cells exists [8, 23], but it requires direct interaction with the code and is not always customizable to the needs of a research question.

In the last decade, artificial intelligence, and particularly deep learning, have gained importance in the analysis of medical image data [27, 14, 22]. Convolutional neural networks (CNNs), a form of deep learning, are particularly well-suited to extract patterns from imaging data [17]. More recently, fueled by the widespread use of high-resolution whole slide imaging of histological sections, CNNs have been applied to the analysis of digitized histopathological data [14]. Their use can facilitate the count of cells or other histological objects [29]. CNNs have also previously been trained to recognize and quantify CAA and Aβ-plaques [31]. However, the concurrent assessment of further markers (e.g. hemorrhagic or inflammatory) in the same data set is of importance to assess complex disease associations.

**Fig. 1:**
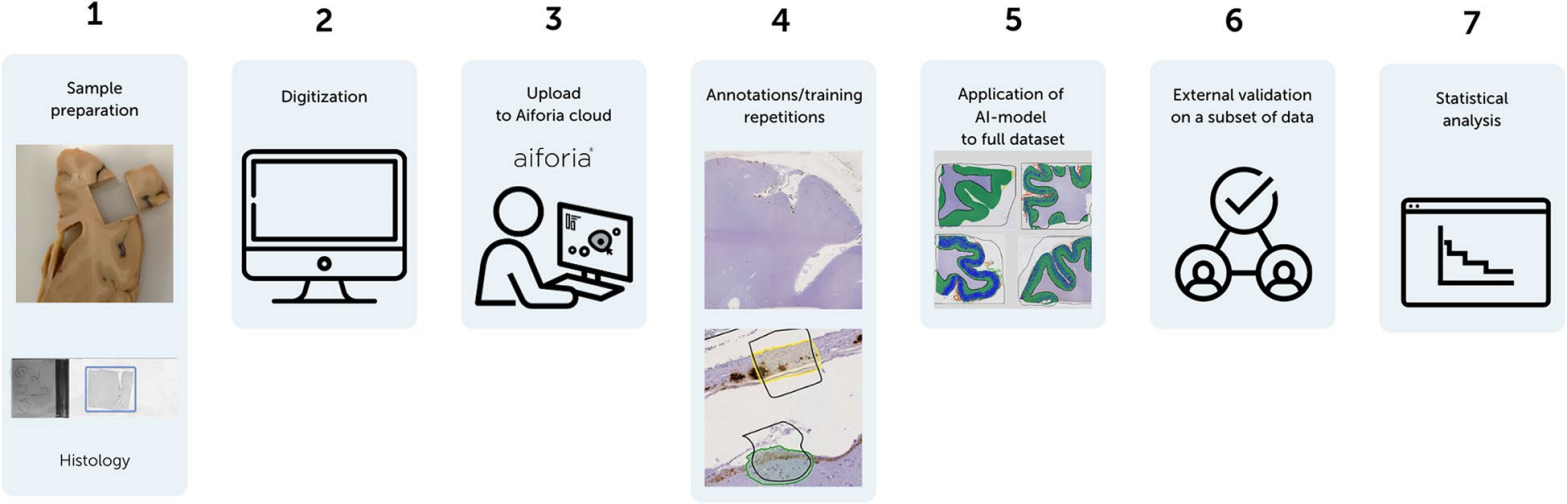
Workflow for development of histopathological deep learning-based models. (1) Preparation and staining of histopathological tissue sections; (2) Digitization of high resolution whole slide images; (3) Uploading on the Aiforia ® cloud based platform; (4) Annotations on a representative subset (approximately 10%) of the whole dataset and repetitive training of the separate convolutional neural networks (CNNs), that constitute the deep learning-based model (AI-model); (5) Application of the model on the whole dataset; (6) Validation of each CNN; (7) Statistical analysis

In this study, we present a workflow (Fig. 1) for training, application, and validation of customizable CNNs, aiming to analyze various histopathological markers common in both AD and CAA. We used Aiforia® Cloud v4.6 (Aiforia Inc., Cambridge, United States), a cloud-based platform, that supports all the steps to build CNNs for histopathological analysis, without requiring the user to have specific technical knowledge about deep learning or programming. Furthermore, we aim to validate the performance of some of the CNNs in relation to previously obtained semiquantitative scores in the same datasets. Finally, we demonstrate that the continuous measures obtained by the CNNs allow building more complex statistical models to assess the interplay of different pathological markers.

## Materials and methods

### Human brain tissue

The deep learning-based models were trained on and applied to digitized histopathological sections derived from available datasets in our lab. Sections from a total number of 47 autopsy cases were included that came from three separate sources: 1) cases with a definite diagnosis of cerebral amyloid angiopathy (CAA) [18] and non-CAA control cases from the neuropathology department at Massachusetts General Hospital [35], 2) Alzheimer’s disease (AD) cases from the South West Dementia Brain Bank at the University of Bristol (REC reference 18/SW/0029), and 3) immunized (as well as placebo-treated) AD cases provided by Profs Boche and Nicoll at the University of Southampton (REC reference 075/03/w).

### Histopathology

Standard sampled formalin fixed tissue blocks were processed, embedded in paraffin, and cut in 6 μm-thick serial sections on a microtome. Immunohistochemistry was performed with antibodies against Aβ (1:200, Dako Cat# M0872), fibrin(ogen) (1:500, Dako Cat# A0080), glial fibrillary acidic protein (GFAP) (1:1,000, Millipore Sigma Cat# G9269), and cluster of differentiation 68 (1:500, Dako Cat# M0814). Briefly, sections were deparaffinized and rehydrated through xylene and graded ethanol series. Next, endogenous peroxidase was blocked with 3% H_2_O_2_ (20 min), followed by formic acid treatment (for Aβ, 5 min) or antigen retrieval in heated citrate buffer (for GFAP, and fibrin(ogen)). Tissue was then blocked with normal horse or goat serum (1 h) and incubated overnight with the primary antibody at 4 °C. On the next day, the biotinylated mouse or rabbit secondary antibody was applied (1 h), followed by a mixture of avidin (A) and biotinylated HRP (B) for 30 min (Vectastain ABC kit, Vector laboratories, 30 min). 3,3'-Diaminobenzidine (DAB, Vector laboratories) was used as the chromogen. After DAB treatment, sections were counterstained with hematoxylin (10 s), dehydrated, and cover slipped with Fisher Chemical Permount mounting medium. Adjacent sections were stained with Luxol fast blue hematoxylin and eosin (LH&E) for myelin, von Kossa for calcium, and Perls’ Prussian blue for iron depositions, following standard histological procedures. The number of sections stained with each method is reported in Table 1.

**Table 1:** Characteristics of AI-models

AI model	Aβ-model	Iron model	Fibrin model	GFAP model	CD68 model	Calcium model
Stain	IHC for Aβ	Perls’ Prussian blue	IHC for Fibrin(ogen)	IHC for GFAP	IHC for CD68	von Kossa
No. of training sections/total no. of sections in dataset	13/146	16/144	13/142	11/144	8/39*	9/44*
Layer 1	*Area*	*Area*	*Area*	*Area*	*Area*	*Area*
	Leptomeningeal vessels	Tissue	Non-vascular tissue	Tissue	Tissue	Tissue
	Tissue		Vascular fibrin positive tissue			
Layer 2	*Area*	*Object*	*Object*	*Object*	*Object*	*Area (Calcium positive)*
	CAA	Iron positive cells	Fibrin positive cells	Reactive astrocytes	CD68 positive cells	Neuronal
	Amyloid-β plaques					Vascular
						Extracellular
Layer 3						*Object*
						Calcium positive cells
No. of sections excluded after QC	4	5	3	0	0	0
No. of sections for validation	13	14	14	14	4	4

The table summarizes the six models described in this study, listing the kind of staining for each set of sections, the number of sections on which the model was trained, and the layers by which the model was built. For each layer it is reported whether the convolutional neural network was trained to recognize an area or an object. Finally, the total number of sections on which the model was applied, those excluded after QC, and those on which the model was validated is reported. *Key:* IHC: immunohistochemistry; AD: Alzheimer’s disease; CAA: cerebral amyloid angiopathy; QC: quality control

^*^Note that the CD68 and calcium models were trained on and applied to sections derived only from cohorts 2 and 3 (AD cases), whereas the other four models used data from all 3 cohorts (AD and CAA cases)

### Training of CNNs

Stained sections were digitized using the NanoZoomer Digital Pathology (NDP)-HT whole slide scanner (C9600-12; Hamamatsu Photonics, Hamamatsu, Japan) with a 20 × objective. The obtained high-resolution (457 nm/pixel; 55,579 dpi) digital whole slide images were then visualized using the NDP.view2 software (version 2.8.24) and next uploaded to the Aiforia® Cloud v4.6 (Aiforia Inc., Cambridge, United States) for image processing (cloud.aiforia.com).

Each deep learning-based model was trained on annotations (labelling) made by AAS and SJvV on a subset of the digital whole slide images (Table 1). Annotations were made using either a drawing tool or an object detector provided by the graphical interface. The subset constituted of approximately 10% of the available sections, which were chosen to ensure capturing the variability in image and staining quality across each dataset.

The models consisted of multiple nested ‘layers’, where each subsequent child ‘layer’ only analyses pixels passed by the previous. The ‘layers’ are independent CNNs that run in a sequential and dependent fashion mimicking human decision making for scoring pathology images. Individual CNNs (or layers) were combined sequentially to create a single model capable of simultaneous detection of tissue areas and objects of interest. CNNs were trained using an increasing number of annotations and iterations, until the model performed satisfactorily. Additional information for the advanced parameters of each CNN, such as image augmentation parameters, perceptive view (field of view), and level of complexity are summarized in Additional file 1: Tables 1 – 6.

**Fig. 2:**
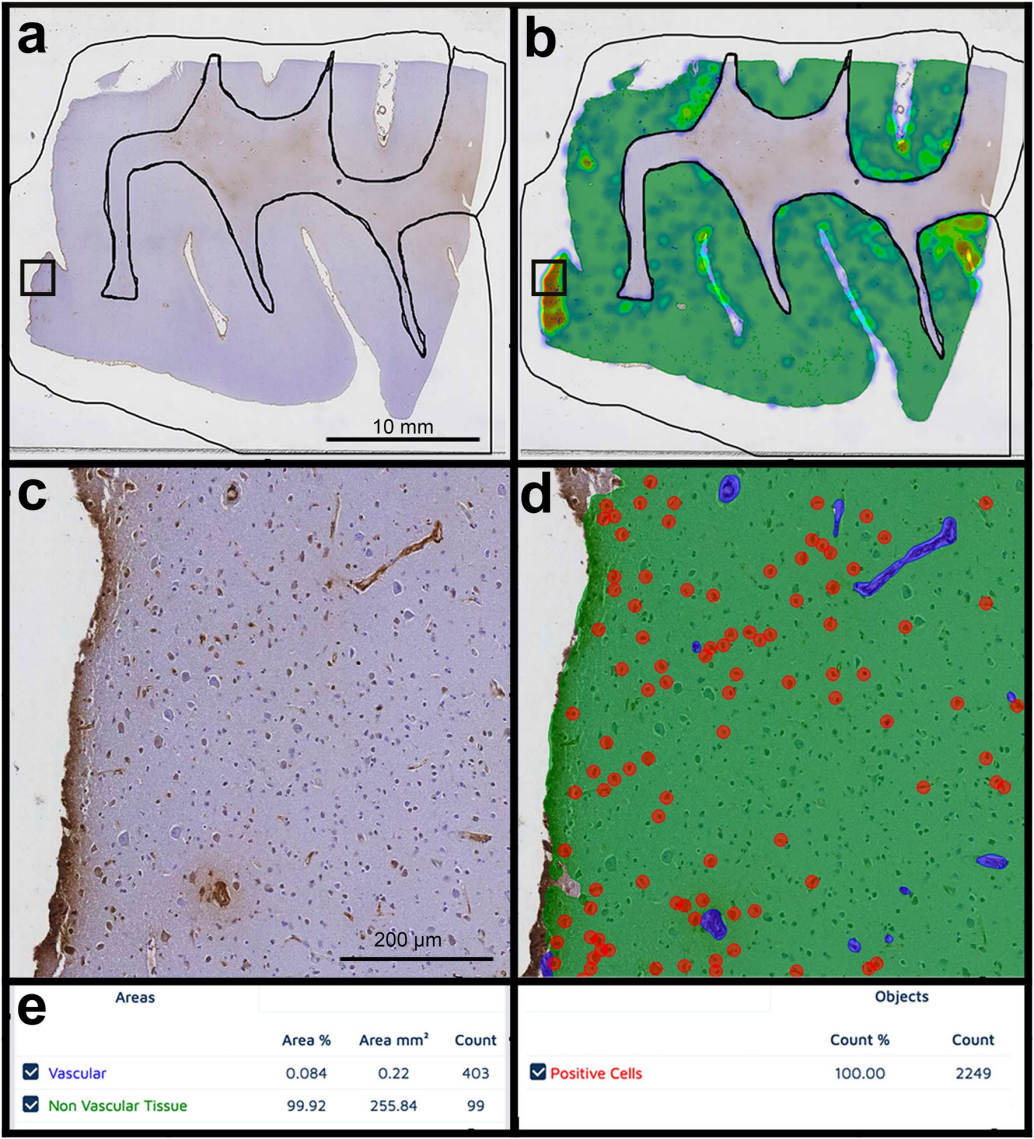
Example of the application of a deep learning-based model (fibrin). Fibrin-stained digitized whole slide section (**a**) and the corresponding heat map for the fibrin model (**b**). Black lines represent the manual segmentation of the cortex. Details are shown respectively in the *insets* (**c**, **d**). The fibrin model was trained to recognize the classes of fibrin positive vascular (blue) and non-vascular tissue (green), as well as fibrin-positive cells (circled in red). Legend of the model as it appears in the Aiforia ® interface is shown (**e**)

CNNs were trained to recognize and quantify areas or objects, depending on the features of interest. Examples of areas are “non-vascular tissue” vs “vascular tissue” or “cortical tissue” vs “leptomeningeal tissue”. Examples of object detection were “iron positive cells” and “fibrin positive cells” (See Table 1 for details and Fig. 2 as an example).

### Deep learning-based models and their output measures

Six separate deep learning-based models were trained to quantify distinct histopathological features common to both AD and CAA (Table 1 and Additional file 1: Tables 1, 2, 3, 4, 5, 6).

1. *Aβ model:* Percentage area of leptomeningeal CAA (leptomeningeal CAA area [mm^2^]/leptomeningeal tissue area [mm^2^]); percentage area of CAA (cortical CAA area [mm^2^]/cortical tissue area [mm^2^], WM CAA area [mm^2^]/WM tissue area [mm^2^]); percentage area of Aβ-plaques (cortical Aβ-plaques area [mm^2^]/cortical tissue area [mm^2^], WM Aβ-plaques area [mm^2^]/WM tissue area [mm^2^]).
2. *Iron model:* Cortical density of iron positive cells (number of iron positive cells/cortical tissue area [no/mm^2^]).
3. *Fibrin model:* Percentage area of fibrin positive vascular tissue (fibrin positive vascular tissue area [mm2]/(cortical tissue area [mm2] + fibrin positive vascular tissue area [mm2])); density of fibrin positive cells (number of fibrin positive cells/tissue area [mm^2^]). Measures were calculated for both cortex and WM.
4. *GFAP model:* Cortical density of reactive astrocytes (number of GFAP positive cells/cortical tissue area [no/mm^2^]).
5. *CD68 model:* Density of activated microglia (number of CD68 positive cells/tissue area [no/mm^2^]). Measures were calculated for both cortex and WM.
6. *Calcium model:* Percentage of calcium positive tissue area (total calcium positive area [mm^2^]/tissue area [mm^2^]); percentage of calcium positive vascular tissue area (calcium positive vascular area [mm^2^]/tissue area [mm^2^]); percentage of calcium positive extracellular area (calcium positive extracellular tissue area [mm^2^]/tissue area [mm^2^]); percentage of calcium positive cellular area (calcium positive cellular tissue area [mm^2^]/tissue area [mm^2^])). Measures were calculated for both cortex and WM.

### Application of CNNs

Once trained, the CNNs were applied respectively to all available sections. To obtain results for cortical grey matter (GM) and white matter (WM), sections were first manually segmented into regions of interest, guided by the adjacent LH&E-stained sections, which provides excellent contrast between GM and WM.

All sections were then visually inspected to evaluate CNN performance. Firstly, quality control was performed at 1 × magnification to assess tissue recognition. Sections that showed more than 5% mislabeling of tissue were excluded from further analysis. Secondly, two randomly chosen areas of the section were inspected at 20 × magnification and the respective section excluded if the objects or area classification within the zoomed in region was erroneous for more than 5% (Table 1).

### Validation of CNNs

**Fig. 3:**
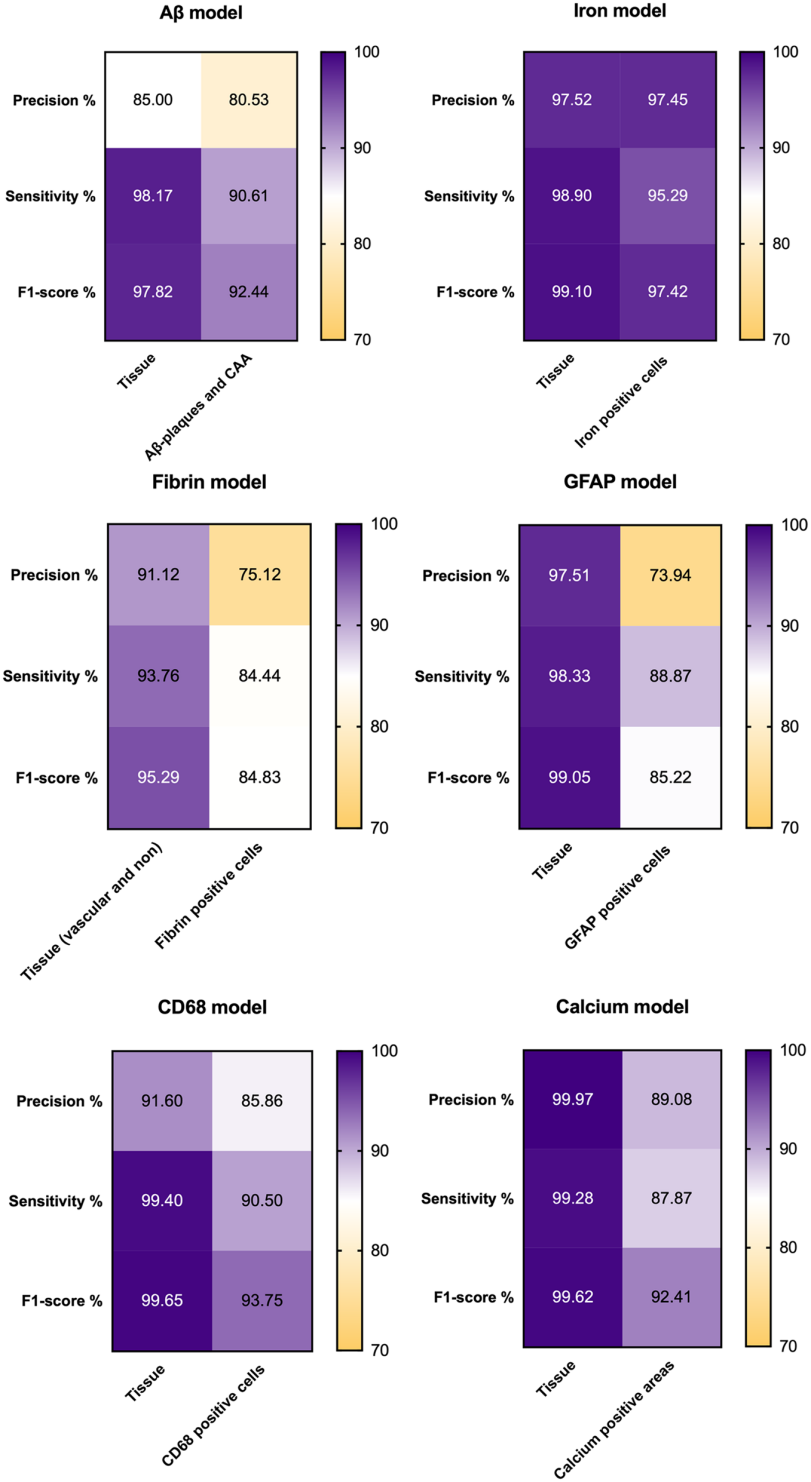
Heatmaps for validation measures of deep learning-based models. The heatmaps show validation measures for all deep learning-based models, with precision, sensitivity and F1-score (rows) for each convolutional neural network (CNN) (column) calculated as an average of all regions of interest and all three validators. These values represent therefore the performance of the model compared to the ground truth (i.e. all external validators). *Key:* False positives (FP), false negatives (FN), precision (TP/[TP + FP]), sensitivity (TP/ [TP + FN]) and F1-score (2 × Precision x Sensitivity/[Precision + Sensitivity])

Next, validation of all CNNs was performed on an independent test set constituted by a subset of sections (approximately 10% of digital whole slide images), different from those on which the model had been trained. Ten validation regions per layer, per section were drawn by VP. Within these regions, the marker of interest was annotated by three independent human validators (SJvV, AAS, and MGK). The percentage of false positives (FP), false negatives (FN), precision (TP/[TP + FP]), sensitivity (TP/[TP + FN]) and F1-score (2 × Precision x Sensitivity/[Precision + Sensitivity]) for each CNN versus each human validator were obtained for all validation regions and subsequently averaged across all validators. To determine the overall performance of each model, these measures were then averaged across the three validators (Fig. 3). The same measures were calculated to evaluate the performance among validators.

### Semiquantitative scores

The reliability of the Aβ and iron models were assessed by comparing the AI-assisted quantitative measures (percentage area of cortical and leptomeningeal CAA, percentage area of cortical parenchymal Aβ-plaques, and density of cortical iron positive cells) with previously obtained semiquantitative scores [10, 31]. For this purpose, a subset of 68 sections were used, derived from a total number of 17 CAA and non-CAA cases (7 females, 10 males; mean age at death [standard deviation] 76.53 [10.10] years) from the MGH cohort (see [11] for more details). These sections included standard sampled areas from the frontal, parietal, temporal, and occipital lobes.

The digitized histopathological sections were visually assessed using the NDP.view2 software (version 2.8.24). Cortical and leptomeningeal CAA severity respectively were evaluated on Aβ-stained sections using a 4-point scale: absent (0), scant Aβ deposition (1), some circumferential Aβ (2), and widespread circumferential Aβ (3) [24]. Similarly, degree of cortical Aβ-plaques was assessed using a 4-point scale as absent (0), mild (1), moderate (2), and severe (3) [21]. Presence and severity of iron-positive deposits in the cortical layers were determined on Perls’ Prussian blue-stained sections as absent (0), mild (1), moderate (2), and severe (3). For these scores, consensus was achieved or inter-rating performed, as previously reported [11]. Within the CAA cases (n = 13 cases, total number of 52 sections), we used a linear mixed effects (LME) model to assess the relationship between cortical and leptomeningeal CAA and density of iron-positive cells in the cortex.

### Association between reactive astrocytes and Aβ pathologies in CAA

Next, the association between reactive astrocytes, Aβ-plaques, and cortical CAA was assessed within a subset of the CAA cases from the MGH cohort (n = 13 cases, total number of 52 sections) [11]. The analysis was performed using an LME model.

### Statistical analysis

Statistical analyses were performed using the software R, version 3.6.0 (R Foundation for Statistical Computing, Vienna, Austria; www.R-project.org) and the Statistical Package for Social Science (IBM SPSS Statistics), version 25. Significance was set at p < 0.05 and all values were two-tailed. Spearman rank-order correlation coefficient was calculated between CNN-derived measures (percentage of cortical and leptomeningeal CAA area, percentage of Aβ-plaque area, and density of iron-positive cells) and semiquantitative scores.

We then used linear mixed effects models (LME), with the R-package “lme4” version 1.1–26 [4], to test whether leptomeningeal and/or cortical CAA were a predictor of density of iron-positive cells in the cortex. Subject and cortical region (frontal, temporal, parietal, and occipital) were defined as random factors, in this way accounting for subject and region dependent differences in pathology. A second LME model was adopted, to assess the association between Aβ-plaques, cortical CAA, and reactive astrocytes. Fixed factors were age at death, sex, percentage of cortical CAA area and percentage of Aβ-plaque area, whereas subject and cortical region were set as random factors. Cortical density of GFAP-positive cells was the dependent variable.

## Results

### Performance of deep learning-based models

Visual quality control was completed on all sections on which the models were applied and showed an excellent performance of the GFAP (Additional file 1: Fig. 1), CD68, and calcium models. Based on our pre-specified exclusion criteria, no section needed to be excluded in these models. Misclassification of > 5% tissue or one of the markers of interest resulted in exclusion of four, five, and three sections in respectively the Aβ, the iron, and the fibrin model (Table 1). Hence, the number of excluded sections remained below 5% for each dataset.

All CNNs were validated according to the annotations performed by three independent validators on predefined validation regions. Precision, sensitivity, and F1-score were good (> 80%) to excellent (> 90%) for all CNNs (Fig. 3), except for GFAP- and fibrin-positive cells, which showed a precision of 73.94% and 75.12% respectively. This can be attributed to a relatively high number of GFAP and fibrin positive cells that were identified by the CNN, but not consistently classified as reactive astrocytes by the raters (false positives). Validators showed similar levels of agreement among each other as compared with the CNNs (Additional file 1: Fig. 2).

### Association between visual semiquantitative scores and deep learning-derived measures

**Fig. 4:**
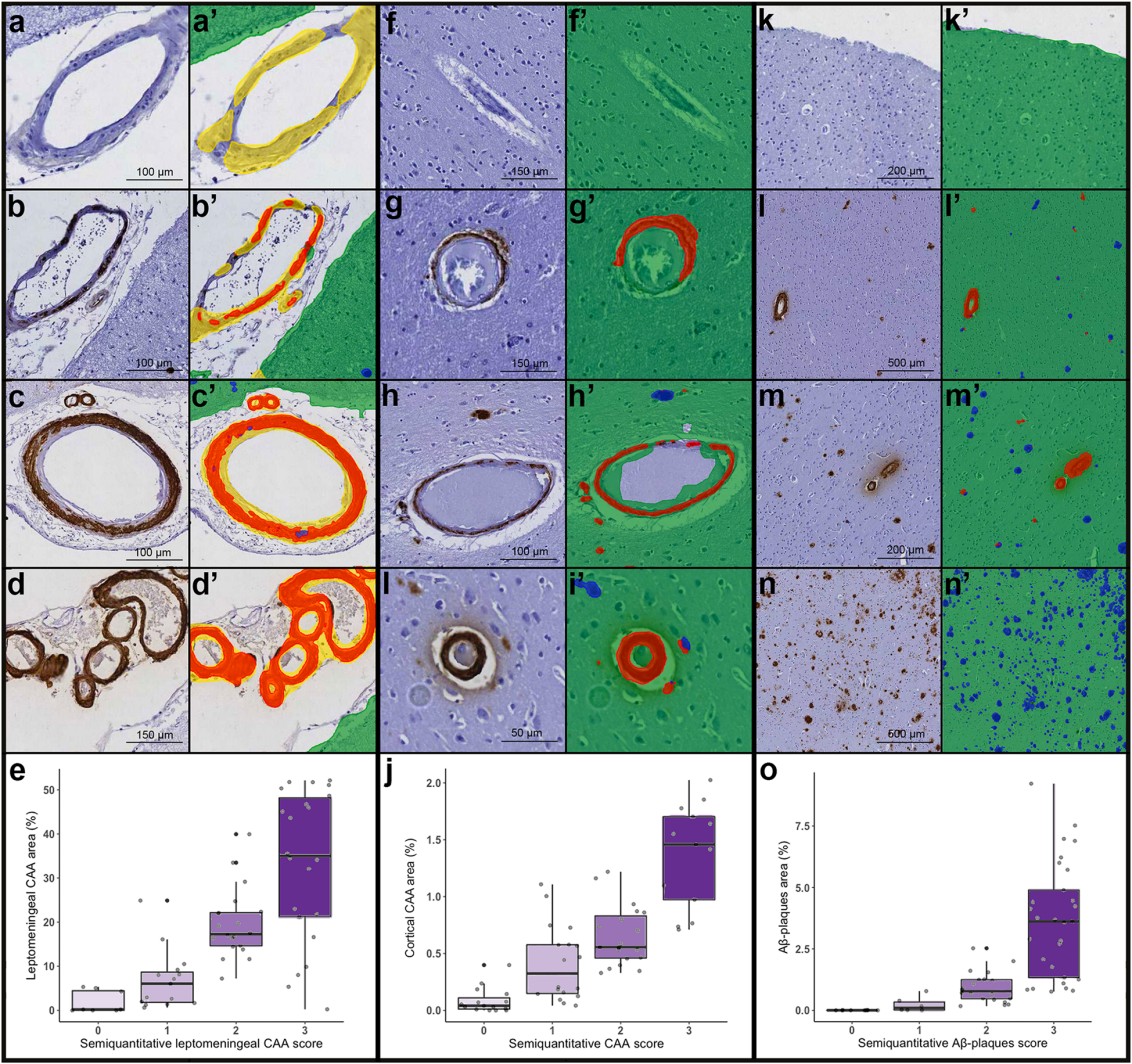
Correlation between semiquantitative scores and deep learning-derived measures for the amyloid-β model. Leptomeningeal vessels differentially affected by CAA are shown (**a** absent; **b** mild; **c** moderate; **d** severe) together with the related deep learning-derived prediction (**a**’–**d**’). Similarly, representative cortical vessels with different degrees of CAA accumulation are shown (**f** absent; **g** mild; **h** moderate; **i** severe) together with the related deep learning-derived prediction (**f**’-**i**’). Finally, degrees of Aβ-plaque severity are shown (**k** absent; **l** mild; **m** moderate; **n** severe) together with the deep learning-derived prediction of the same area (**k**’–**n**’) Box plots show the correlation between semiquantitative visual scores obtained in a total number of 65 whole slides for leptomeningeal CAA (**e**), cortical CAA (**j**), and Aβ-plaques (**o**) and the respective measure obtained using the deep learning-model. Interquartile range (top and bottom of the box), median (central band), outliers (data points beyond the whiskers), and individual data points are visualized. *Key:* green = cortical tissue; yellow = leptomeningeal tissue; red = CAA; blue = Aβ-plaques

**Fig. 5:**
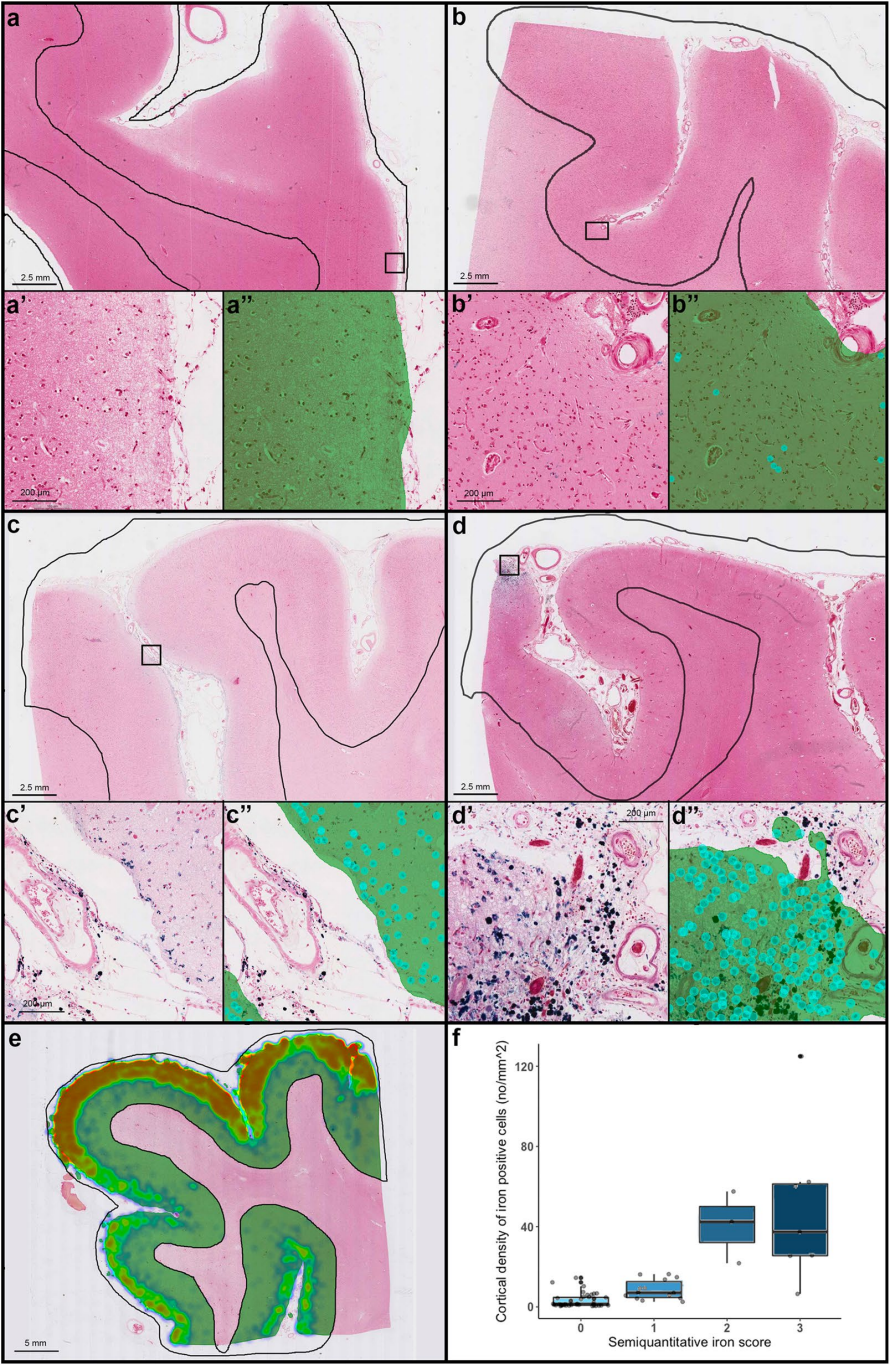
Iron model and correlation with the respective semiquantitative score. Severity of cortical iron deposits is shown in the overview (**a** absent; **b** mild; **c** moderate; **d** severe) and in greater detail in the *insets*, which reveal iron-positive cells (in blue) (**a**’–**d**’). **a**’’–**d**’’ show the respective deep learning-derived prediction. Within the manually segmented cortex (black lines in **a**–**d**), tissue area recognized by the model is overlaid in green, while objects (iron-positive cells) are identified by light blue circles. Iron-model heat map of a whole slide from a CAA case with cortical superficial siderosis (**e**). Box plots show the correlation between semiquantitative visual scores and continuous measures derived using the deep learning-based model in a total number of 65 whole slides. Interquartile range (top and bottom of the box), median (central band), outliers (data points beyond the whiskers) and individual data points are visualized. *Key:* green = cortical tissue; light blue = iron-positive cells

After visual quality control, three of the 68 sections were excluded for calculations concerning the Aβ model and three from those concerning the iron model (see above). Semiquantitative scores (0–3) of cortical and leptomeningeal CAA strongly correlated with deep learning-derived measures of cortical and leptomeningeal CAA percentage area (cortical CAA: ρ = 0.82, p < 0.001; leptomeningeal CAA: ρ = 0.75, p < 0.001) (Fig. 4). Semiquantitative scores (0–3) of cortical Aβ-plaques were also strongly associated with deep learning-derived Aβ-plaques percentage area (ρ = 0.84, p < 0.001) (Fig. 4). Moreover, semiquantitative scores (0–3) of iron deposits strongly correlated with the density of iron positive cells identified by the AI (ρ = 0.72, p < 0.001) (Fig. 5).

The few discrepancies between the results of the CNNs and the semiquantitative scores were visually inspected and could be explained by either human error, higher sensitivity of the deep learning-based model, or patchy distribution of the pathology (Additional file 1: Fig. 3).

Next, we used the newly obtained continuous measures from the iron and Aβ models which reproduced the previously reported relationship between leptomeningeal CAA severity and degree of cortical superficial siderosis in a clinical CAA cohort [11]. Using an LME model, the density of iron-positive cells in the cortex was associated with the percentage of leptomeningeal CAA area (β = 0.64; 95% confidence interval (CI) [0.08—1.150]; p = 0.014), but not percentage of cortical CAA area (β = -6−29; 95% confidence interval (CI) [−18.21—5.65]; p = 0.278), confirming previous observations derived from semiquantitative scores. The total LME model’s R^2^ was 0.49.

### Cortical CAA is associated with density of reactive astrocytes

Finally, we explored the relationship between reactive astrocytes and disparate types of Aβ pathologies, using an LME model. Cortical CAA percentage area (β = 7.28; 95% confidence interval (CI) [− 0.71–14.80]; p = 0.059), but not Aβ-plaques area (β = − 1.36; 95% confidence interval (CI) [− 3.81–1.23]; p = 0.260) tended to be associated with the density of reactive astrocytes in the cortex. This exploratory finding suggests that accumulation of Aβ in the walls of blood vessels rather than the parenchyma may result in neuroinflammation in the form of reactive astrocytes. The total LME model’s R^2^ was high (0.81).

## Discussion

In this study, we presented a deep learning-based workflow to obtain quantitative measures of common histopathological features of AD and CAA. The cloud-based platform Aiforia ® enabled us to build six deep learning-based models to identify and quantify the two hallmark Aβ pathologies of AD and CAA (Aβ-plaques and vascular Aβ-deposition), as well as other histopathologic alterations that play a role in the pathophysiology of both neurodegenerative diseases. The models enabled us to produce objective, continuous measures for density of iron-positive cortical cells, which have been identified in AD [7, 11] and which represent the pathological correlate of cortical superficial siderosis, a characteristic neuroimaging marker of CAA [11, 34]. Furthermore, we were able to identify GFAP positive cells (a marker of reactive astrocytes) and CD68 positive cells (a marker of activated microglia), which are both indicative of neuroinflammation [3, 20, 26]. The activation of neuroinflammatory pathways has increasingly been recognized as a key element involved in AD and CAA [18, 33] and may be a response to BBB leakage [16]. The fibrin model detects and quantifies fibrin positive cells and vessels as a measure of plasma protein extravasation from the blood vessels and is thus an indicator of BBB leakage [11, 15, 23]. The final model targeted calcium positive areas, which have previously been observed in the hippocampus of AD patients [30] and within the context of severe CAA [7].

The performance of the CNNs based on precision, specificity and F1-score was very good (> 80%) to excellent (> 90%) for all models. Validators showed similar measures of agreement between each other and with the deep learning-based models. Exceptions were precision for the identification of GFAP and fibrin positive cells (73% and 75% respectively). Both indicate a lower positive predictive value, due to relatively high number of false positives: non-astrocytic GFAP positive cells for the former and capillaries for the latter. Further optimization of these models may therefore be warranted prior to applying them to additional (external) datasets. The deep learning-derived measures were consistent with experts’ observations for leptomeningeal and cortical CAA, Aβ-plaques, and iron-positive cells, as it has previously been shown with similar approaches [29]. These results represent an incentive to use these deep learning-derived measures in future studies.

In general, deep learning-based models can help to upgrade the pathological staging and grading of CAA and Aβ-plaques. Scales that assess severity of CAA [1, 24] and Aβ-plaques [21] pathology on single histopathological sections could benefit from such models that increase precision (e.g. using percentage area of the tissue occupied by the pathology of interest, rather than semiquantitative scores). Even when the scoring protocols are carefully described and inter-rating performed, they can be misinterpreted and often leave some room for subjectivity (e.g. does the score report the CAA/Aβ-plaques averaged severity of the whole section or the most severe pathology found?). Furthermore, while the harmonization of a semiquantitative score requires time-consuming consensus meetings among experts [24], the performance of an AI-model could be easily and objectively evaluated across cohorts, thus increasing feasibility of harmonized multicentric studies. Moreover, the description of neuropathological stages of AD based on the topographical distribution of Aβ-plaques [13] and neurofibrillary changes [6] might benefit from adding a more quantitative approach to the existing descriptive ones. The distinction between different pathological subtypes of Aβ-plaques (e.g. diffuse, neuritic, dense core, and the newly described coarse-grained plaques) [5] and CAA (e.g. capillary CAA) [22, 35] has proven feasible with CNNs [29] and can also be achieved through our workflow. Given that different types of Aβ-plaques may reflect different mechanisms [28], their differential quantification could provide more insight into the disease pathophysiology. The added value of using deep learning is the possibility to incorporate many different aspects of pathological phenotyping (different forms of proteinopathy, quantification of their severity, their subtypes, and topographical distribution) within a single analysis.

The availability of continuous measures allows the use of more complex statistical models that depend on continuous outcome variables, such as LME models, which can combine a greater number of predictors than the more limited approaches applicable to discontinuous categorical variables. For example, we assessed the relationship between density of cortical iron-positive cells and CAA using an LME model. In this way, we reproduced the previously observed association between leptomeningeal CAA and iron-positive cells (i.e. the histopathological correlate of cortical superficial siderosis) [11], while also controlling for cortical CAA as a potential confounder. As suggested before, these findings confirm the notion that cortical superficial siderosis is the result of chronic bleeding form leptomeningeal blood vessels with severe CAA rather than cortical blood vessels, which are significantly less affected in CAA cases with siderosis. Adopting a similar model, we were able to investigate the association between reactive astrocytes and two disparate manifestations of Aβ deposition within the same brain area: parenchymal vs. vascular Aβ. Interestingly, we found that reactive astrocytes were more strongly associated with cortical CAA compared to Aβ-plaques. This is in line with the notion that astrocytes play an important role in regulating the BBB [9] and the neurovascular unit [15], which are both disrupted in the context of severe vascular Aβ deposition. Similar approaches of neuropathological analysis could benefit the precise localization and quantification of microglia, pericytes, and other cell types and thus better understand the role these cells play in neuroinflammatory responses in individuals with coexisting AD and CAA pathologies.

In summary, the use of deep learning in the assessment of histopathological markers has several advantages over traditional approaches. First, it provides more objective measures than semiquantitative scores. Second, the availability of continuous measures enables the application of more complex statistical models, such as LME models, that allow to account for a greater number of factors. Third, after the successful training of a CNN, this approach is more sustainable when compared with the alternative time-consuming rating or counting and can thus potentially facilitate harmonization of histopathological analysis across cohorts. Finally, the use of an online cloud-based platform such as Aiforia ®, offers the possibility to researchers without specific knowledge in deep learning to train and validate CNNs for histopathological analysis.

A limitation of this study includes the use of a proprietary software that is not open source. That said, the models described in this study are available within the Aiforia ® platform to other investigators who seek to analyze similar datasets. These models offer advantages that are particularly valuable for statistical analysis and thus for research purposes, whereas their use and potential benefit for clinical neuropathological diagnostic has not been evaluated here.

## Conclusion

To the best of our knowledge, this is the first study to present a variety of deep learning-based-models, able to identify and quantify several biologically relevant histopathological markers of AD and CAA. Most importantly, the chosen workflow is easy for any researcher with pathological expertise to implement, provides objective quantitative measures, and is customizable for further markers and research questions. In conclusion, the application of deep learning in general opens new avenues for the use of histopathology in the study of neurodegenerative disease and facilitates harmonization across datasets and centers.

## Supplementary Information

**Additional file 1.** Advanced parameters

## Data Availability

Performance of the Aβ model on a representative subset of histopathological sections can be evaluated here: https://cloud.aiforia.com/usermanagement/addviewertogroup?urlguid=02da4075-469f-4fbf-b010-d4f718a93f37 (password: AZ_CAA_CNN). All deep learning-based models described in this study will be made available within the Aiforia ® platform to other investigators upon reasonable request. Finally, the source data have been deposited here: https://dataverse.harvard.edu/dataset.xhtml?persistentId=doi:10.7910/DVN/UJWTN2.
