# Supplementary material for: Deep learning assisted quantitative assessment of histopathological markers of Alzheimer’s disease and cerebral amyloid angiopathy
Source: Acta Neuropathol Commun. 2021 Aug 21;9:141. doi: 10.1186/s40478-021-01235-1 (PMC8380352; doi:10.1186/s40478-021-01235-1)
Supplement: Supplementary file 1 — Additional file 1. Advanced parameters [file 40478_2021_1235_MOESM1_ESM.docx]

**Supplementary Information:**

**Deep learning assisted quantitative assessment of histopathological markers of Alzheimer’s disease and cerebral amyloid angiopathy**

Valentina Perosa MD^1,2*^, Ashley A. Scherlek BM^3,4*^, Mariel G. Kozberg MD PhD^4^, Lindsey Smith^5^, Thomas Westerling-Bui^5^, Corinne A. Auger BM^4^, Serge Vasylechko PhD^6^, Steven M. Greenberg MD PhD^1^, Susanne J. van Veluw PhD^1,4^

*^1^J. Philip Kistler Stroke Research Center, Department of Neurology, Massachusetts General Hospital, Harvard Medical School, Boston, MA, USA*

*^2^ Department of Neurology, Otto-von-Guericke University, Magdeburg, Germany*

*^3^Rush Alzheimer Disease Center, Rush University Medical Center, Chicago, IL, USA*

*^4^ MassGeneral Institute for Neurodegenerative Disease, Massachusetts General Hospital, Charlestown, MA, USA*

*^5^ Aiforia Inc, Cambridge, MA, USA*

*^6^ Computational Radiology Laboratory, Boston Children's Hospital, Boston, MA, USA*

* Both authors contributed equally to this work.

Corresponding author:

Valentina Perosa, MD

J. Philip Kistler Stroke Research Center, Massachusetts General Hospital

Cambridge Str. 175, Suite 300

Boston, MA 02114, USA

Tel: +1 8572631663

Fax: +1 [617-726-5043](tel:617-726-5043)

[VPEROSA@mgh.harvard.edu](mailto:VPEROSA@mgh.harvard.edu)

The advanced parameters applied to each model are reported in the tables below. These parameters are constituted by i) training parameters for each layer, that are meaningful for training of regions and/or objects, and ii) image augmentation parameters, which define the degree of variability added to the data during the training.

1. **Training parameters:**

- *Field of view (μm):* Size of the view needed to have a minimum context around the pattern in order to recognize the feature (for region).
- *Complexity (simple, intermediate, complex):* Parameter that indicates the degree of difficulty in distinguishing the layer-related pattern from the rest of the image (for region and object). ‘Model Complexity’ is a preset combination of several parameters that have been shown to work best for different types of digital pathology tasks, the parameters change both model depth (number of layers) and width (number of nodes in a layer).
- *Weight decay:* Constant parameter used when training convolutional neural networks, in order to prevent the weights to grow too large and thus overfitting of the model (for region and object).
- *Mini-batch size:* Size of the batches (in number of annotations) in which the training data set is split during the iterations (for region and object).
- *Mini-batch per iteration:* number of mini-batches involved in each iteration (for region and object).
- *Iterations without progress:* Number of iterations the network goes through without learning anything new (for region and object).
- *Initial learning rate:* Learning rate value of the neural network (for region and object).
- *Window size (px):* size of the region, that contains the object of interest (for objects).
- *Maximum object overlap (%):* Percentage of area overlap between two objects, at which the model recognizes a single object.

1. **Image augmentation parameters**: variability (in percentage) is added to the parameters listed below.

- *Scale*
- *Aspect ratio*
- *Maximum shear*
- *Luminance*
- *Contrast*
- *Maximum white balance change*
- *Noise*

|  | **Layer 1**  **Cortical and leptomeningeal tissue** | **Layer 2**  **CAA and amyloid-β plaques** |
| --- | --- | --- |
| **Field of View (μm)** | 100 | 30 |
| **Complexity** | Complex | Very complex |
| **Weight decay** | 0.0001 | 0.0001 |
| **Mini-batch size** | 80 | 40 |
| **Mini-batches per iteration** | 20 | 20 |
| **No. iterations without progress** | 1000 | 1000 |
| **Initial learning rate** | 0.1 | 0.1 |
| **Training window size, min-max (px)** | 256-2048 | 256-2048 |
| **Maximum object overlap (%)** | N/A | N/A |
| **Scale (max/min)** | -1/1.01 | -1/1.01 |
| **Aspect Ratio (%)** | 1 | 1 |
| **Maximum Shear (%)** | 1 | 1 |
| **Luminance, max/min (%)** | -1/1 | -1/1 |
| **Contrast, max/min (%)** | -1/1.01 | -1/1.01 |
| **Max. white balance change** | 1 | 1 |
| **Noise** | 0 | 0 |

**Supplementary Table 1: Advanced parameters for the amyloid-β model.** Key: N/A = not applicable.

|  | **Layer 1**  **Tissue** | **Layer 2**  **Iron positive cells** |
| --- | --- | --- |
| **Object size (μm)** | N/A | 22 |
| **Field of View (μm)** | 125 | 110.5 |
| **Complexity** | Intermediate | Intermediate |
| **Weight decay** | 0.0001 | 0.0001 |
| **Mini-batch size** | 120 | 350 |
| **Mini-batches per iteration** | 20 | 20 |
| **No. iterations without progress** | 1000 | 1000 |
| **Initial learning rate** | 0.1 | 0.1 |
| **Training window size, min-max (px)** | 256-2048 | 200 |
| **Maximum object overlap (%)** | N/A | 95 |
| **Scale (max/min)** | -10/10 | -1/1.01 |
| **Aspect Ratio (%)** | 10 | 1 |
| **Maximum Shear (%)** | 10 | 1 |
| **Luminance, max/min (%)** | -10/10 | -1/1 |
| **Contrast, max/min (%)** | -10/10 | -1/1.01 |
| **Max. white balance change** | 5 | 1 |
| **Noise** | 2 | 0 |

**Supplementary Table 2: Advanced parameters for the iron model.** Key: N/A = not applicable.

|  | **Layer 1**  **Non-vascular and fibrin positive vascular tissue** | **Layer 2**  **Fibrin positive cells** |
| --- | --- | --- |
| **Object size (μm)** | N/A | 22 |
| **Field of View (μm)** | 75 | 110.5 |
| **Complexity** | Complex | Intermediate |
| **Weight decay** | 0.0001 | 0.0001 |
| **Mini-batch size** | 80 | 350 |
| **Mini-batches per iteration** | 20 | 20 |
| **No. iterations without progress** | 500 | 500 |
| **Initial learning rate** | 0.1 | 0.1 |
| **Training window size, min-max (px)** | 256-2048 | 200 |
| **Maximum object overlap (%)** | N/A | 95 |
| **Scale (max/min)** | -1/1.01 | -1/1.01 |
| **Aspect Ratio (%)** | 1 | 1 |
| **Maximum Shear (%)** | 1 | 1 |
| **Luminance, max/min (%)** | -1/1 | -1/1 |
| **Contrast, max/min (%)** | -1/1.01 | -1/1.01 |
| **Max. white balance change** | 1 | 1 |
| **Noise** | 0 | 0 |

**Supplementary Table 3: Advanced parameters for the fibrin model.** Key: N/A = not applicable.

|  | **Layer 1**  **Tissue** | **Layer 2**  **GFAP positive cells** |
| --- | --- | --- |
| **Field of View (μm)** | 100 | 192.9 |
| **Complexity** | Intermediate | Intermediate |
| **Weight decay** | 0.0001 | 0.0001 |
| **Mini-batch size** | 120 | 350 |
| **Mini-batches per iteration** | 20 | 20 |
| **No. iterations without progress** | 500 | 500 |
| **Initial learning rate** | 0.1 | 0.1 |
| **Training window size, min-max (px)** | 256-2048 | 256 |
| **Maximum object overlap (%)** | N/A | 95 |
| **Scale (max/min)** | -10/10 | -10/10 |
| **Aspect Ratio (%)** | 10 | 10 |
| **Maximum Shear (%)** | 10 | 10 |
| **Luminance, max/min (%)** | -10/10 | -10/10 |
| **Contrast, max/min (%)** | -10/10 | -10/10 |
| **Max. white balance change** | 5 | 5 |
| **Noise** | 2 | 2 |

**Supplementary Table 4: Advanced parameters for the GFAP model.** Key: N/A = not applicable.

|  | **Layer 1**  **Tissue** | **Layer 2**  **CD68 positive cells** |
| --- | --- | --- |
| **Object size (μm)** | N/A | 25 |
| **Field of View (μm)** | 100 | 251.1 |
| **Complexity** | Intermediate | Intermediate |
| **Weight decay** | 0.0001 | 0.0001 |
| **Mini-batch size** | 120 | 350 |
| **Mini-batches per iteration** | 20 | 20 |
| **No. iterations without progress** | 1000 | 1000 |
| **Initial learning rate** | 0.1 | 0.1 |
| **Training window size, min-max (px)** | 256-2048 | 400 |
| **Maximum object overlap (%)** | N/A | 95 |
| **Scale (max/min)** | -10/10 | -10/10 |
| **Aspect Ratio (%)** | 10 | 10 |
| **Maximum Shear (%)** | 10 | 10 |
| **Luminance, max/min (%)** | -10/10 | -10/10 |
| **Contrast, max/min (%)** | -10/10 | -10/10 |
| **Max. white balance change** | 5 | 2 |
| **Noise** | 2 | 0 |

**Supplementary Table 5: Advanced parameters for the CD68 model.** Key: N/A = not applicable.

|  | **Layer 1**  **Tissue** | **Layer 2**  **Calcium positive regions (extracellular, vascular)** | **Layer 3**  **Calcium positive cells** |
| --- | --- | --- | --- |
| **Object size (μm)** | N/A | N/A | 22 |
| **Field of View (μm)** | 100 | 40 | N/A |
| **Complexity** | Intermediate | Very complex | Intermediate |
| **Weight decay** | 0.0001 | 0.0001 | 0.0001 |
| **Mini-batch size** | 120 | 40 | 350 |
| **Mini-batches per iteration** | 20 | 20 | 20 |
| **No. iterations without progress** | 1000 | 1000 | 1000 |
| **Initial learning rate** | 0.1 | 0.1 | 0.1 |
| **Training window size, min-max (px)** | 256-2048 | 256-2048 | 256-2048 |
| **Maximum object overlap (%)** | N/A | N/A | 95 |
| **Scale (max/min)** | -10/10 | -1/1.01 | -1/1.0 |
| **Aspect Ratio (%)** | 10 | 1 | 1 |
| **Maximum Shear (%)** | 10 | 1 | 1 |
| **Luminance, max/min (%)** | -10/10 | -1/1 | -1/1 |
| **Contrast, max/min (%)** | -10/10 | -1/1 | -1/1 |
| **Max. white balance change** | 5 | 1 | 1 |
| **Noise** | 2 | 0 | 0 |

**Supplementary Table 6: Advanced parameters for the calcium model.** Key: N/A = not applicable.

**
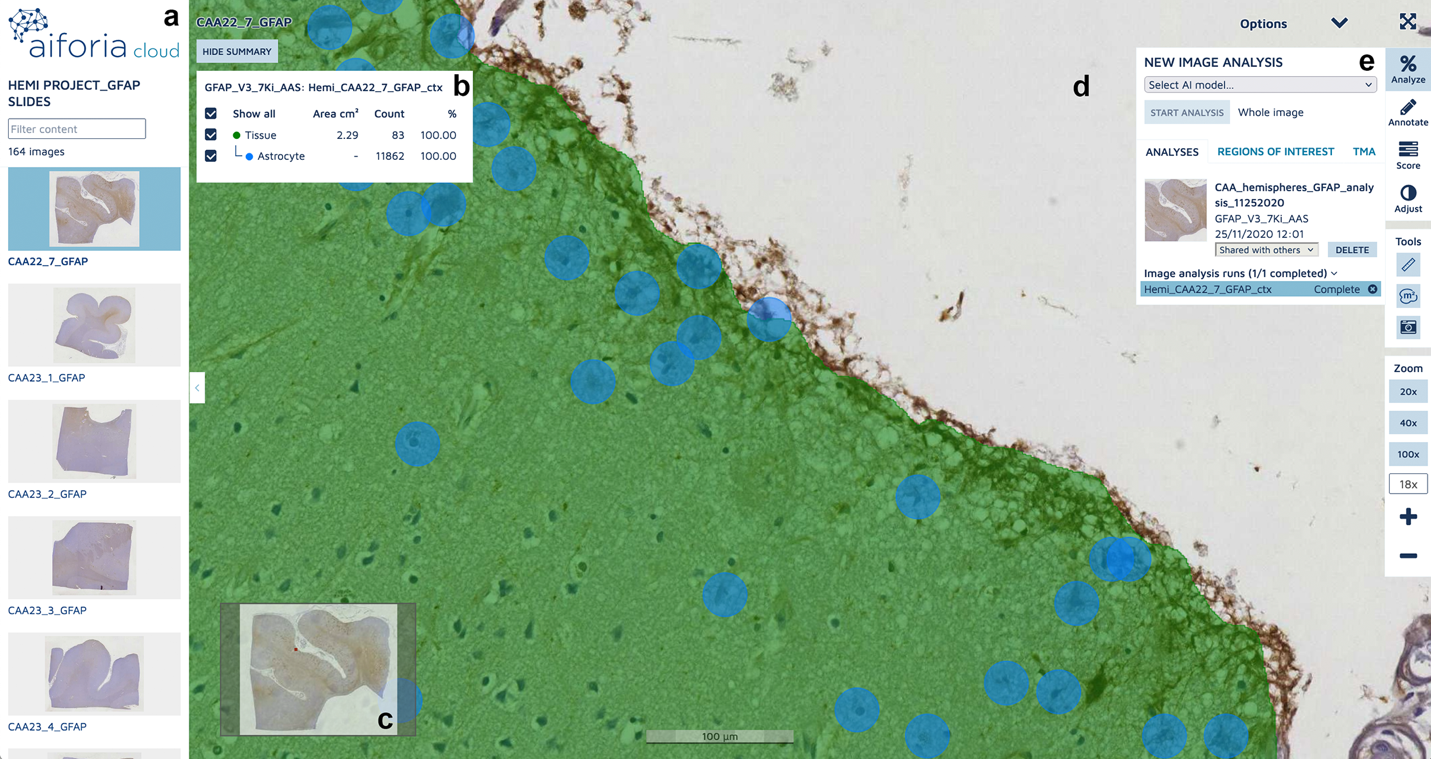
**

**Supplementary Fig. 1 Graphical interface on the Aiforia® platform.** The screenshot of the Aiforia® platform environment when visualizing results of the GFAP model. Thumbnails of the GFAP stained sections included in the dataset a). Legend of the model showing the two layers, that constitute the GFAP-model b). Overview of the whole slide section with zoomed-in area outlined with a red box c). Detail of the selected section, in which tissue (green) and reactive astrocytes (blue) can be recognized d). Panel of options, which include the image analysis on a specific region of interest (e.g. the cortex) e).

**
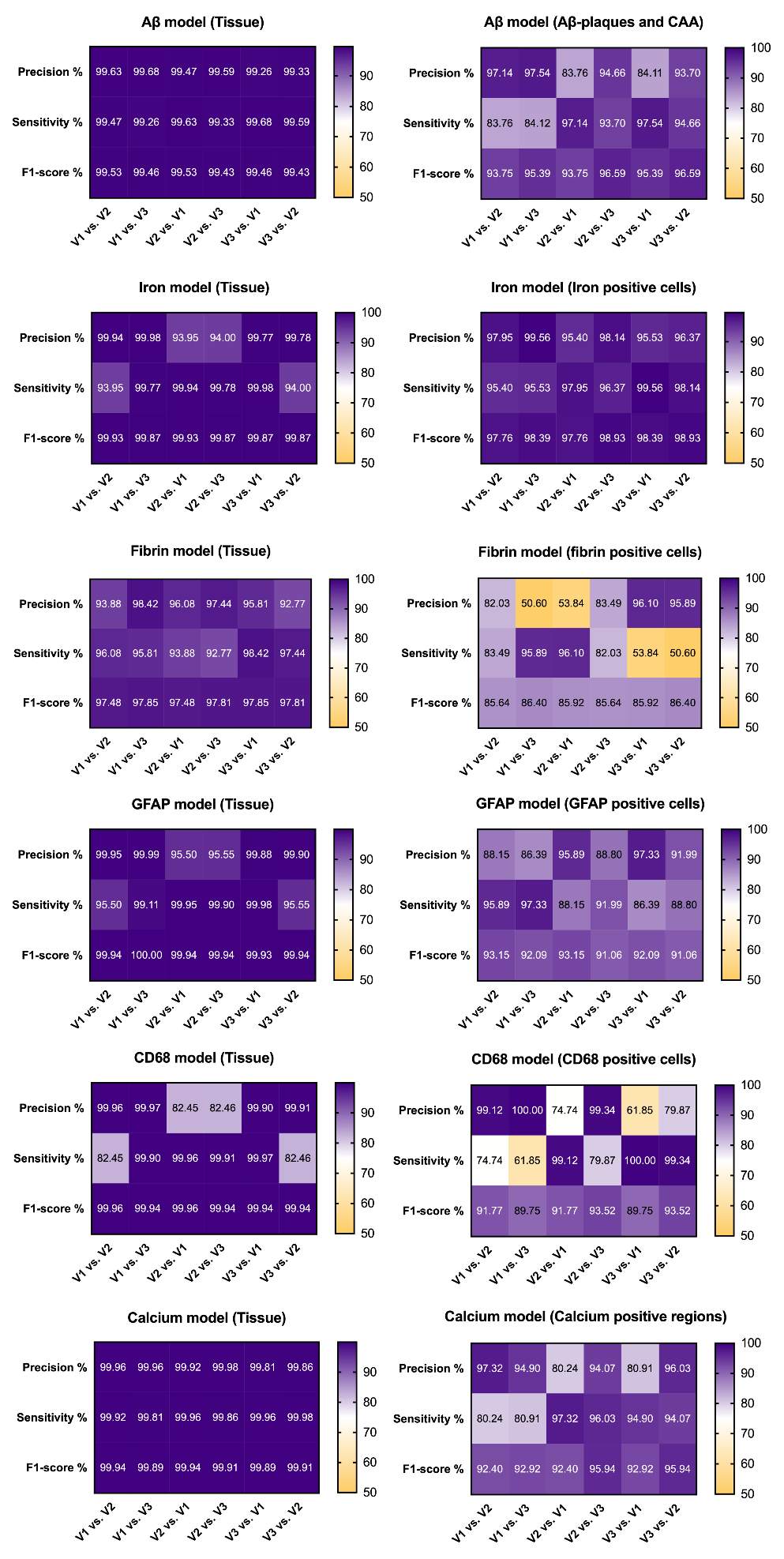
**

**Supplementary Fig. 2 Heat maps for validation results of all validators and all deep learning-based models.** The heat maps show validation measures for all deep learning-based models, with precision, sensitivity, and F1-score (rows), setting each validator against another validator’s annotations (columns). Measures are calculated as an average of all regions of interest. Each heat map represents results for a different CNN (layer). *Key:* False positives (FP), false negatives (FN), precision (TP/[TP+FP]), sensitivity (TP/ [TP +FN]), and F1-score (2 x Precision x Sensitivity/[Precision + Sensitivity])

**
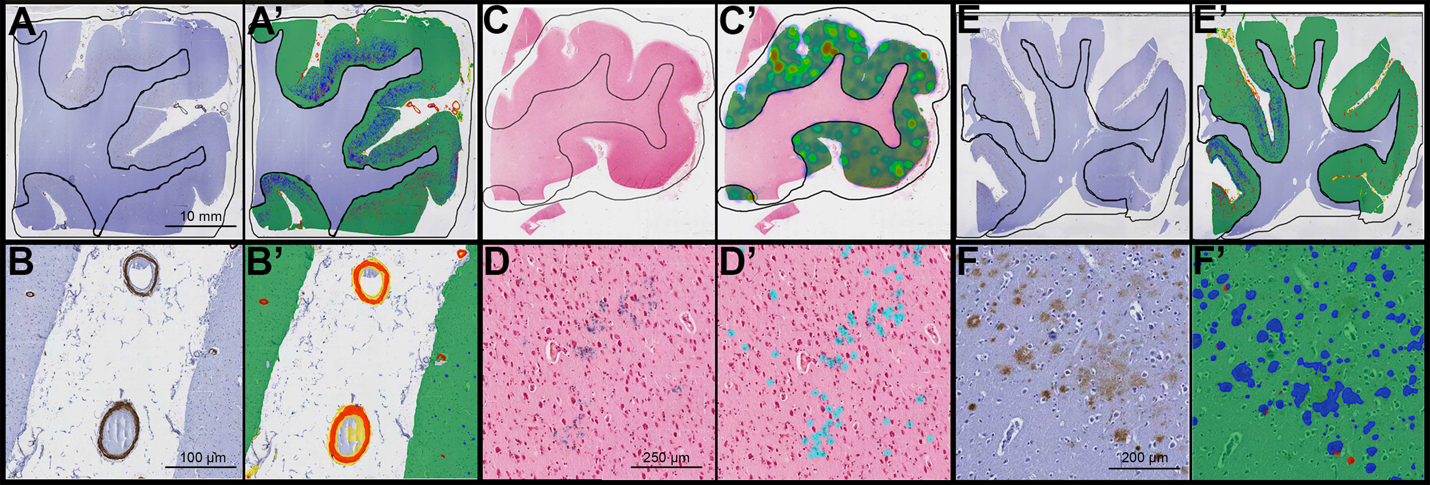
**

**Supplementary Fig. 3** **Examples of discrepancies between the visual semiquantitative scores and AI-model detection.** Example of an amyloid-β (Aβ) stained section with low semiquantitative score (0), but relatively high leptomeningeal CAA area (25%) (a, corresponding heat map in a’). At visual inspection, the AI-model estimated the severity of leptomeningeal CAA correctly (circumferential deposition), as shown in the insets (b, b’). Example of a Perls’ Prussian blue stained section, with low semiquantitative score (0), but relatively high density of iron positive objects (15.3 no/mm^2^) (c, and corresponding heat map c’). Closer inspection revealed that some blue staining artefacts were wrongly recognized as iron positive objects by the AI-model (d, d’). In some cases, (e and corresponding heat map e’), both the semiquantitative visual score and the AI correctly estimated the severity of the pathology. The apparent discrepancy between the high visual semiquantitative score (3) for the Aβ-plaques and the relatively low percentage area measured by the AI-model, can be explained by the patchy distribution of the plaques.
